# Organoids as tools to investigate gastrointestinal nematode development and host interactions

**DOI:** 10.3389/fcimb.2022.976017

**Published:** 2022-08-12

**Authors:** Ruby White, Frances Blow, Amy H. Buck, María A. Duque-Correa

**Affiliations:** ^1^ Institute of Immunology & Infection Research, School of Biological Sciences, University of Edinburgh, Edinburgh, United Kingdom; ^2^ Cambridge Institute of Therapeutic Immunology and Infectious Disease, University of Cambridge, Cambridge, United Kingdom

**Keywords:** organoid, gastrointestinal nematodes, gastric epithelium, intestinal epithelium, host-parasite interactions, immunomodulation, nematode life cycles

## Abstract

Gastrointestinal nematodes are a diverse class of pathogens that colonise a quarter of the world’s human population and nearly all grazing livestock. These macroparasites establish, and some migrate, within host gastrointestinal niches during their life cycles and release molecules that condition the host mucosa to enable chronic infections. Understanding how helminths do this, and defining the molecules and mechanisms involved in host modulation, holds promise for novel strategies of anthelmintics and vaccines, as well as new knowledge of immune regulation and tissue repair. Yet the size and complexity of these multicellular parasites, coupled with the reliance on hosts to maintain their life cycles, present obstacles to interrogate how they interact with the gastric and intestinal epithelium, stroma and immune cells during infection, and also to develop protocols to genetically modify these parasites. Gastrointestinal organoids have transformed research on gastric and gut physiology during homeostasis and disease, including investigations on host-pathogen interactions with viruses, bacteria, protozoa and more recently, parasitic nematodes. Here we outline applications and important considerations for the best use of organoids to study gastrointestinal nematode development and interactions with their hosts. The careful use of different organoid culture configurations in order to achieve a closer replication of the *in vivo* infection context will lead not only to new knowledge on gastrointestinal nematode infection biology, but also towards the replication of their life cycles *in vitro*, and the development of valuable experimental tools such as genetically modified parasites.

## Introduction

The gastrointestinal (GI) tract is a complex enclosed system composed of multiple organs that, together with commensal microbes, is responsible for the digestion and absorption of nutrients and for the protection against incoming pathogens. Pathogen infections impact many aspects of GI function and thus can contribute to disease; however, it has been challenging to model infection processes *in vitro*. Organoids systems have greatly improved the ability to study the GI epithelium *in vitro*: by culturing either induced pluripotent stem cells or isolated tissue adult stem cells this technology successfully recapitulates the key features of the *in vivo* epithelium (Puschhof et al., 2021). Organoids have been used to identify new interactions of the GI epithelium with a plethora of microparasites, including viruses (e.g. enabling the culture of norovirus for the first time) (Ettayebi et al., 2016), bacteria (e.g. *Helicobacter pylori*, *Escherichia coli*) (McCracken et al., 2014; Pleguezuelos-Manzano et al., 2020), and protozoa (e.g. *Cryptosporidum parvum*) (Heo et al., 2018). Organoid cultures have helped build a deeper understanding of these infections, and their links to disease such as cancer, and these applications have been reviewed extensively elsewhere (Bartfeld, 2016; Dutta and Clevers, 2017; Dutta et al., 2017; Barrila et al., 2018; Duque-Correa et al., 2020a; Puschhof et al., 2021).

**Figure 1:**
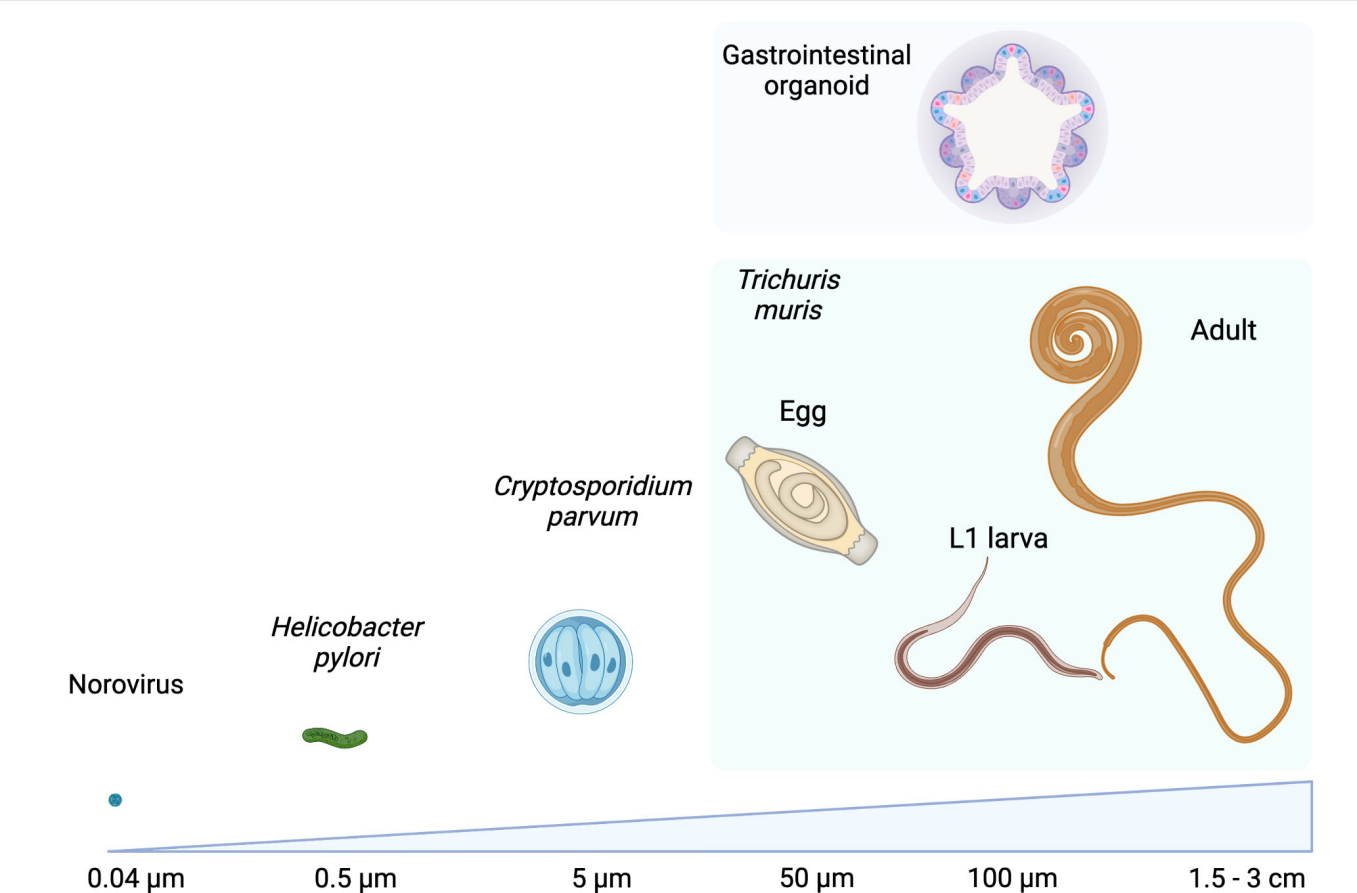
Comparison of sizes of examples of microorganisms that have been co-cultured with GI organoids with those of different life stages of the GI nematode *Trichuris muris* alongside a GI organoid. Values refer to the approximate diameter or length of each organism, images not to scale. Created with BioRender.com.

The GI epithelium is also a niche for macroparasites, including GI nematodes, which infect a quarter of the world’s human population and all grazing livestock (Loukas et al., 2016; Jourdan et al., 2018; Charlier et al., 2020; Else et al., 2020). GI nematodes are multicellular pathogens that establish chronic infections, resulting in modifications of the GI epithelium that could be driven by the parasites and their excretory/secretory (ES) molecules, or indirectly by host immune responses to infection (Coakley and Harris, 2020). While there is good understanding of the immune drivers of epithelial modification during GI nematode infection, the direct actions of the parasites and their products on the epithelium remain largely unknown. To date, this research has mostly used animal infection models (Coakley and Harris, 2020; Duque-Correa et al., 2020a; Baska and Norbury, 2022), in which teasing out the direct impact of nematodes on the epithelium is difficult. Moreover, for the majority of human infective GI nematodes, no animal host systems are available, which has limited our ability to directly study nematode-host interactions in these species. Organoids are a promising methodology to overcome these barriers and to provide a mechanistic understanding on the effects of these parasites on their host niches. However, using organoids in GI nematode research is not trivial since GI nematodes are generally 10-10,000 fold larger than viruses, bacteria and protists (**Figure 1**) and have longer, and in some cases intricate, life cycles where the parasites interact with multiple cells and organs. In this Perspective, we detail how the study of GI nematode-epithelial interactions has interfaced with development of organoids and describe the potential applications of these systems for advancement of GI nematode research.

## Organoids as models of GI nematode niches

GI nematodes have a tropism for specific organs of the GI tract of their host e.g. whipworms colonise the caecum and proximal colon, hookworms and threadworms establish in the small intestine, while sheep GI parasites find a niche in the abomasum. *In vivo* the epithelium of these organs/regions vary greatly in their cell type content, mucus layer composition and thickness, size, architecture, and expression of toll-like receptors, and these features are replicated to some extent by their respective organoid cultures (Mowat and Agace, 2014; Price et al., 2018; Duque-Correa et al., 2020a; Kayisoglu et al., 2021).

Traditional organoid cultures use basement membrane extracts (BME), which mimic the laminin rich extracellular matrix, alongside the supplementation of key growth factors and morphogens that are specific to the host and organ of origin, and allow for the division and differentiation of stem cells (reviewed elsewhere) (Sato and Clevers, 2013; Beumer and Clevers, 2020; Hou et al., 2020). These cultures result in self-organised three-dimensional (3-D) structures that display cellular polarisation, with the apical surface of the epithelium facing inwards towards the lumen (basal-out), and that are capable of differentiating into multiple cell types of the epithelium of origin (Kayisoglu et al., 2021; Puschhof et al., 2021). Apical-out organoids are an alternate approach to 3-D organoid cultures that allows access to the apical surface of the epithelium. Apical-out organoids are generated by mechanical disruption of traditional 3-D organoids followed by suspension culture in the absence of BME. This procedure induces the inversion of the polarity and repair of the epithelium, thus the apical membrane faces outwards, while the organoid 3-D structure is maintained (Co et al., 2019; Co et al., 2021).

Organoids can also be cultured in a 2-D conformation by dissociating 3-D organoids into single cell suspensions that are seeded into either cell culture plates, or semi-permeable membranes (tranwells) coated with BME, laminin or collagen (Moon et al., 2014; Aguilar et al., 2021). 2-D organoids maintain cellular polarisation and show a degree of crypt-like spatial organisation, albeit to a lesser extent than 3-D cultures. 2-D organoids grown on semi-permeable membranes create a model with physically separated apical and basal culture compartments, which allows for greater control over delivery of growth factors, cytokines, pathogens and other cellular populations, to either the basal or apical membrane of the epithelium (Moon et al., 2014; Kozuka et al., 2017; Duque-Correa et al., 2020a; Aguilar et al., 2021; Puschhof et al., 2021). 2-D organoids grown on semi-permeable membranes also enable longer culture lengths than 3-D models with recent studies extending culture for up to 2 months (Boccellato et al., 2019).

Recently, more advanced technologies that allow for enhanced recapitulation of *in vivo* structure and physiology have been developed through the use of micro-engineered scaffolds or microfluidic chips (termed organ-on-a-chip) (Hofer and Lutolf, 2021). Micro-engineered scaffolds constrain the architecture of growing organoids to physical boundaries that mimic *in vivo* structures dimensions. For example, using collagen scaffolds, enteroids successfully replicated crypt and villus architecture, and allowed for long-term culture for up to 4 weeks (Wang et al., 2017; Nikolaev et al., 2020). Micro-engineered scaffolds and other microchip formats are also capable of reproducing additional physiological aspects not present in 3-D or 2-D organoids, such as a fluid flow rate, shear forces, stimulation of peristalsis, automated nutrient supply and waste removal (Kasendra et al., 2018; Nikolaev et al., 2020; Yin et al., 2021). For instance, vascularisation can be mimicked using microchips containing multiple microcompartments: the lower compartment is seeded with endothelial cells while the upper is seeded with 3-D human enteroid cells. In this system, the presence of underlying endothelium and a fluid flow rate enhanced the differentiation of the epithelium (Kasendra et al., 2018; Yin et al., 2021).

The different dimensional conformations in which organoids can be cultured could be exploited to model the diverse interactions of GI nematodes with their host during invasion and colonisation of tissues, and as systems to investigate nematode modification of the epithelium and host immunomodulation during infection. In the next sections, we will discuss important considerations on the use of organoids to investigate these biological processes.

## Unravelling GI nematode invasion using organoids

Infection of the GI tract by nematodes occurs via ingestion/swallowing of parasite eggs or infective larvae. The first crucial step for GI nematodes to establish a successful infection is the sensing by the parasites of a suitable environment to hatch and/or invade the host epithelium (Mkandawire et al., 2022). The specific cues, signalling pathways and mechanisms behind these processes are currently not well defined, but represent a key stage for intervention in the transmission of these parasites.

Organoids are an attractive model for understanding GI nematode invasion because they recapitulate many physicochemical and cellular characteristics of the *in vivo* host niche that may promote infection. Moreover, organoids could allow real time visualisation of invasion dynamics, which cannot be investigated using animal models. For example, murine caecaloids reproduce the mucus layer and cellular composition of the caecal epithelium and successfully promote the epithelium invasion and formation of syncytial tunnels by *Trichuris muris* first-stage (L1) larvae *in vitro* (Duque-Correa et al., 2022).

When developing organoid models to study GI nematode invasion, it is critical to consider which surface of the epithelium the parasites are in contact with when interacting with their hosts *in vivo*. For GI nematodes that invade apically, modelling invasion using 3-D organoids is difficult due to the large size of the parasites (**Figure 1**). Smaller organisms such as viruses, bacteria or protists can be delivered to traditional (basal-out) 3-D organoids by microinjection, or by shearing of the organoids followed by co-incubation (Aguilar et al., 2021; Puschhof et al., 2021). However, microinjection is not suitable for delivery of any life stage of GI nematodes, as even eggs and larval stages are too large for the microinjection needles, and the luminal volume of 3D-organoids is not big enough to host the parasites (Duque-Correa et al., 2020a). Moreover, shearing of 3-D organoids followed by co-incubation with whole parasites is unlikely to result in their successful incorporation into the organoid lumen. An alternative method to deliver live GI nematodes into the lumen of traditional 3-D organoids is their addition to the organoid culture media. This strategy was used to infect both ovine and bovine abomasum organoids with *Teladorsagia circumcinta* and *Ostertagia ostertagi*, respectively (Smith et al., 2021; Faber et al., 2022). Strikingly, *T. circumcinta* and *O. ostertagi* L3 larvae migrated through the BME and transversed the organoid membrane, from the basal to apical side into the lumen (Smith et al., 2021; Faber et al., 2022). L3 larvae of *O. ostertagi* also transversed bovine organoids from the apical to the basal side (Faber et al., 2022). However, as the authors noted, *T. circumcincta* and *O. ostertagi* are not previously described to cross the epithelial barrier during their life cycles, instead they are thought to invade through gastric neck opening of gastric glands (Smith et al., 2021; Faber et al., 2022). Whether transversion of the epithelium occurs *in vivo* or is unique to these cultures is unknown (Faber et al., 2022). Similarly, it is not known if other GI nematodes co-cultured in this fashion can invade the BME and migrate across the epithelium into the organoid lumen.

2-D organoids grown in semi-permeable membrane systems allow for both basal and apical delivery of GI nematodes, which is advantageous for the *in vitro* modelling of invasion by some species. For instance, L3 *Heligmosomoides polygyrus* bakeri larvae burrow from the GI lumen through the epithelium into the submucosa, where they moult twice to reach adulthood; the adult parasites then transverse the epithelium to emerge into the duodenal lumen (Camberis et al., 2003). Therefore, apical delivery would be better suited to studies on invasion of the epithelium by L3 *H. bakeri*, while basal delivery of *H. bakeri* adult parasites could mimic conditions for re-emerging into the lumen.

2-D organoid systems also enable the study of interactions between the mucus layer and GI nematodes. The mucus layer, or layers in the case of the caecum and colon, overlays the epithelial cells and acts as a substantial physical barrier that protects the epithelium from incoming luminal contents, microbiota and pathogens (Herbert et al., 2009; Bergstrom and Xia, 2022). GI nematodes need to transverse the mucus layer(s) to reach the epithelial cells, but the mechanisms used by the parasites are not well understood. On the other hand, the mucus layer could provide uncharacterised cues for parasite egg hatching and larvae invasion. Thus, modelling the mucus barrier in an accessible way is important for investigations of GI nematode invasion. Studying these processes *in vivo* is challenging due to the size of infective larvae and the lack of protocols to generate stably labelled nematodes for *in vivo* imaging (Duque-Correa et al., 2022). For instance, *in vivo* studies on mucus degradation by L1 *T. muris* larvae during invasion of the caecal epithelium are impeded by the small ratio of larvae versus caecal epithelial cells, which dilutes any effects the larvae have on the mucus layer. However, using 2-D transwell caecaloid cultures in which higher numbers of L1 larvae to a smaller surface area can be achieved, degradation of mucus during early infection was detectable (Duque-Correa et al., 2022).

## *In vitro* modelling of epithelial changes and immunomodulation during GI nematode infection

A major focus of GI nematode research is on understanding the impact of parasite ES products on the modulation of host tissues (Maizels et al., 2018). Research in this area has focussed on the effects these molecules on immune cells. However, during GI nematode infection there are significant modifications of the host GI epithelium that result in parasite expulsion, or that promote parasite persistence (Coakley and Harris, 2020). Currently, little is known on the specific interactions and mechanisms by which GI nematodes and their ES molecules alter epithelial cell proliferation and differentiation (Maizels et al., 2018; Duque-Correa et al., 2020a).

Due to the complex cellular make up *in vivo*, teasing apart the direct effects of the parasites and their ES products on the epithelium from those driven indirectly by host immune responses using animal models is challenging. On the other hand, the reductionistic nature of organoid cultures enables the introduction of live parasites, ES products and immune factors in a controlled manner, and thus they allow the dissection of their individual effects on the GI epithelium. For instance, several GI nematode infections, including *H. bakeri*, induce goblet and tuft cell hyperplasia with subsequent increases in mucus and alarmin production that mediate parasite expulsion (Coakley and Harris, 2020; Baska and Norbury, 2022). The goblet and tuft cell hyperplasia is a consequence of host production of the cytokines interleukin (IL) 25, IL4 and IL13 in a feed-forward loop that is driven by tuft cell sensing of nematode infection (Howitt et al., 2016; Von Moltke et al., 2016; Schneider et al., 2018; Luo et al., 2019). In parallel, GI nematodes immunomodulatory molecules act to minimise these responses in order to persist in their hosts. To understand how this is achieved, Drurey et al. added adult *H. bakeri* ES products to the media of traditional basal-out 3-D enteroids and observed this co-culture resulted in suppression of tuft cell differentiation (Drurey et al., 2021). Surprisingly, this effect was maintained when ES treatment was given in combination with IL4 and IL13, indicating that *H. bakeri* ES products conteract the tuft cells hyperplasia driven by the immune response to the parasite despite an overall increase in tuft cell during *in vivo* infection (Gerbe et al., 2016; Howitt et al., 2016; Von Moltke et al., 2016; Drurey et al., 2021). However, because adult H. bakeri parasites reside within the lumen of the small intestine and thus interact with the apical surface of the epithelium, it is unclear how this co-culture approach where the ES products are in contact with the basal membrane, reflects *in vivo* interactions.

Accordingly, the use of organoids to model host– parasite ES product interactions requires careful consideration of epithelium polarity. The apical and basolateral epithelium have different functions partly defined by the differential localisation of proteins including receptors (Weisz and Rodriguez-Boulan, 2009). Therefore, the accessibility to target receptors could influence the detection of functional effects and should ultimately determine how parasites and their products are delivered when designing organoid experiments. Unlike live nematodes, ES products can be delivered into the organoid lumen via microinjection. For example, extracellular vesicles (EVs) derived from *T. muris*, *Ascaris suum* and *Nippostrongylus brasiliensis* have been successfully microinjected into traditional 3-D organoids, replicating parasite interactions with the apical membrane of the host epithelium (Eichenberger et al., 2018a; Eichenberger et al., 2018b; Chandra et al., 2019; Duque-Correa et al., 2020b). However, microinjection is laborious, does not allow control over the volume/dose injected, and requires specialised equipment and training (Duque-Correa et al., 2020a). Multiple studies have administered ES products from GI nematodes to the culture media of 3-D organoids with the assumption that functional molecules will diffuse through the BME and find their target cells (Drurey et al., 2021; Karo-Atar et al., 2021; Faber et al., 2022). Results from these experiments should be cautiously interpreted as depending on the localisation of the particular parasite life stage this method of delivery may not replicate *in vivo* infection context. Alternatively, apical-out organoids could facilitate the replication of interactions of GI nematodes with the apical epithelium and thus, may be used in future studies (Smith et al., 2021).

2-D organoids grown in semi-permeable membranes overcome the limitations of 3-D organoids by allowing a more accurate modelling of the stimuli the epithelial cells encounter during infection. This includes: 1) the controlled (dose and volume) co-culture of nematodes or their ES products with the apical or basal compartment that mimics the interactions of larval and adult stages with the GI epithelium, and 2) the stimulation with cytokines on the basal compartment to replicate interactions with immune cells that can occur before or after exposure to ES products.

To advance our understanding of how nematode ES interacts with the host epithelium and the functional implications of these interactions on underlying cells, it will be beneficial to introduce additional cell types such as immune and stromal cells, or microbiota, alongside organoids (Duque-Correa et al., 2020a; Sasaki et al., 2020; Puschhof et al., 2021). Adding immune or stromal components to 2-D or micro-engineered scaffold organoid cultures at controlled timings could be used to model nematode-host interactions during either primary or secondary infections, or to increase numbers of rare cell types of interest for experimental purposes which are difficult to study *in vivo* due to low numbers.

## Organoids as systems to recreate the life cycles of GI nematodes

While organoids have been used to study interactions of the GI epithelium with parasitic nematodes or their ES products at a specific developmental stage, their application to recreate the life cycle of these parasites has not been explored yet. Developing *in vitro* systems that support a part or the entire life cycle of GI nematodes will enable studies on their developmental biology and basic requirements for moulting. *In vitro* life cycles would also permit real time observation of the behaviour of the parasites while they undergo the transitions between larval and adult stages. Moreover, these models could allow the investigation of the host-nematode interactions across the life cycle of the parasites, not being restricted to any specific life stage. *In vitro* life cycles could also hold particular value in the capacity to genetically engineer parasites, where targeted mutagenesis of specific life stages may be required to achieve genetic modification.

A key advantage of organoids is that they enable studies on GI nematodes with cells derived from their own host. Thus, organoids could overcome barriers in research focused on aspects of the infection that are host-restricted and cannot be replicated using an animal model due to significant interspecies differences (Duque-Correa et al., 2020a). This is particularly relevant for human GI nematodes that do not have any system for investigation besides controlled infections that have limited experimental read outs (Diemert et al., 2018; Alabi et al., 2021; Pritchard et al., 2021). The lack of models for human infective nematodes has driven the use of nematode parasites of rodents or other mammals, which have similar life cycles or induce comparable pathogenesis as human infective species. Specifically, *T. muris* as a natural whipworm of mice serves as a model of *T. trichiura* (Klementowicz et al., 2012; Else et al., 2020). The rodent hookworms *H. bakeri* and *N. brasiliensis* do not model the full life cycle of any significant human pathogen but have similar life cycles to *Ancylostoma ceylanicum* and *A. caninum* and *Necator americanus*, respectively (Camberis et al., 2003; Bouchery et al., 2017). Moreover, *Trichinella spiralis*, *Ascaris suum* and *T. suis* infect pigs, and *T. vulpis*, *A. duodenale*, *A. ceylanicum* and *A. caninum* can infect dogs, making pigs and dogs viable animal models for the study of GI nematodes (Jenkins, 1970; Hendrix et al., 1987; Gottstein et al., 2009; Pittman et al., 2010; Dold and Holland, 2011; Shepherd et al., 2018). However, the use of large mammals as animal models has considerable limitations including the requirement for specialised housing facilities, associated cost of maintaining these animals, ethical considerations, and the limited ability to track host-parasite interactions over the course of infection in these models.

The recreation of the life cycle of human and livestock GI nematodes *in vitro* using organoids could revolutionise the helminthology field by allowing not only a better understanding of their pathogenesis but also of their “beneficial” effects on the control of inflammation. Organoids could become a key tool to interpret the results of controlled hookworm and whipworm human infections (Diemert et al., 2018; Alabi et al., 2021; Chapman et al., 2021) and on the identification of parasite ES molecules produced by specific larval/adult stages that mediate anti-inflammatory actions of GI nematodes (Maizels et al., 2018). Moreover, organoids could also have a great impact on the development of new anthelmintics and on studies of anthelmintic resistance by allowing investigations on the effects of drugs on the different life cycle stages. Overall, organoids would allow a reduction and, in some cases, a complete replacement of the use of animals in GI nematode research.

## Considerations and challenges on the development of *in vitro* life cycles of GI nematodes using organoids

The life cycles of GI nematodes range from those that are simple, usually taking place in one organ upon ingestion, to complicated ones, involving migration through different tissues (Bouchery et al., 2017). While “body-on-a-chip” technologies are being developed, they are still in their infancy, thus the complete *in vitro* modelling of complicated life cycles, such as those of hookworms (*N. americanus*, *A. duodenale*, *N. brasiliensis*), roundworms (*Ascaris* spp) and threadworms (*Strongyloides stercoralis*, *S. venezuelensis* and *S. ratti*) is not foreseeable soon. In contrast, GI organoids, and organ-on-a-chip technologies could serve as systems to recreate a part (limited to the stages that take place in the GI tract) or the complete life cycle of some GI nematodes.

*In vitro* models supporting the life cycle of these worms would need to replicate the key host features and interactions that serve as cues for the invasion of tissues, growth and moulting of the parasites. Some limitations of current organoids systems to reproduce such cues are their lack of vascularization and innervation. Moreover, while some of the interactions of the epithelium with other cells present in the organ and microbiota can be recreated by the co-culture of organoids with immune and stromal cells and few bacterial species, the full complexity of the tissue is far from being modelled.

To enable the growth and moulting of GI nematodes, organoid cultures should provide an area/volume that accommodates the different life stages, which can range from a few hundred micrometres in the eggs and infective stages up to centimetres in the adults of *Ascaris* spp. In addition, to support life cycle transitions organoid systems sustaining long-term culture would be required as life cycles can take from days to months.

Among GI nematodes, the recreation of the life cycle of *T. spiralis* in enteroids should be the most straightforward, as already Caco-2 cells sustain L1 invasion, moulting, ecdysis, development to adulthood and reproduction of the parasite in only 4-6 days of culture (Gagliardo et al., 2002). Because organoids better replicate the intestinal epithelium than Caco-2 cells, this system would allow an enhanced understanding of the mechanisms used by *T. spiralis* to establish and moult in its intra-multicellular niche.

Like T. spiralis, *T. trichiura* and *T. muris* inhabit multi-intracellular epithelial burrows but in the caecum and proximal colon of their hosts (Else et al., 2020). We have recently developed an *in vitro* model using murine caecaloids grown in transwells that recapitulates the early events of infection by *T. muris* L1 larvae (Duque-Correa et al., 2022). We are currently using this model to further recreate the life cycle of *T. muris*, which *in vivo* takes around 32 days. We have maintained whipworm-infected caecaloids for several weeks and preliminary data indicate that this system supports growth and moulting of whipworms (personal communication). These results would indicate that the interactions of whipworm larvae with the caecal epithelium are sufficient to support parasite development. However, optimisation of this model is still required to improve the numbers of whipworms reaching late larval stages potentially by removing accumulating mucus and dead cells and preventing the exit of larvae from their syncytial tunnels during moulting.

Though *H. bakeri* is a rodent parasite, it has enormously contributed to the knowledge of Type 2 immunity, and research on its immunomodulatory products and their potential therapeutic effect on inflammatory diseases is an area undergoing intense study (McSorley and Maizels, 2012; Maizels et al., 2018; Maizels, 2020). Therefore, the development of an *in vitro* life cycle for this parasite would greatly benefit those investigations. However, there are challenges to creating such a model. First, the requirement of infective L3 larvae to ex-sheath in the stomach before infecting the small intestine (Camberis et al., 2003), which suggests the need of both gastric and intestinal organoids for an *in vitro* system. Second, the creation of complex models mimicking not only the epithelia but also the gastric and small intestinal mucosa and including an outer muscularis layer of the small intestine, where the L3 larvae migrate to ex-sheath and moult to reach adulthood, respectively (Camberis et al., 2003; Bouchery et al., 2017).

Finally, vascularization of organoids or supplementation with blood or its components would be required in an *in vitro* system sustaining GI nematodes that feed from blood such as hookworms and *Haemonchus contortus* (Roeber et al., 2013; Loukas et al., 2016; Ehsan et al., 2020).

## Outlook

The advancement of organoid technologies in the last decade has synergised with increased interest and research on the mechanisms by which GI nematodes interact and manipulate gastric and intestinal epithelia during their life cycles. A particular strength of organoids is that they enable the direct effects of these multicellular parasites and their ES products on the epithelium to be dissected and uncoupled from the changes that result to this tissue due to the immune response to infection. Already from research using organoids in the last years we have learned how GI nematodes and their products degrade mucus during invasion of the epithelia and can directly impact stem cell differentiation and epithelial cell signalling altering their epithelial niches. These findings reveal the value in integrating studies of GI nematodes and their immune modulatory products with those of gut physiology and stem cell biology. Needed future advances on organoid platforms include the incorporation additional cells from the stem cell niche (immune, stromal and neural cells) and the modelling of parameters that impact physiology and function of this tissue, which may also be targeted by the parasites. Further cross-disciplinary initiatives combining parasitology with stem and epithelial cell biology will be pivotal for the advancement of *in vitro* systems that can replicate the *in vivo* host-parasite interactions and life cycles of GI nematodes. These concerted efforts could reveal new signalling mechanisms relevant to multiple disease contexts, and could also help pinpoint the reliance of GI nematodes on host physiology, potentially pointing to new strategies for anthelmintics (most of which were developed more than 50 years ago and show mounting issues with resistance). Finally, a still un-tapped application of organoids is in the ability to maintain and track individual parasites for extended periods, across multiple life stages. Ultimately, this ability could enable screening and selection of parasites for genetic modification, a missing platform on the research on GI nematodes.

## Data availability statement

The original contributions presented in the study are included in the article/supplementary material. Further inquiries can be directed to the corresponding author.

## Author contributions

RW wrote sections of the manuscript regarding the use of organoids as models of GI nematode niches and contributed to the formatting and referencing of the text. FB created the figure accompanying the manuscript and contributed to the article Introduction. AB supported the writing of the introduction and outlook sections. MAD-C contributed to the writing and editing of all sections of the manuscript. All authors edited the article and approved the submitted version.

## Funding

MD-C is supported by a Sir Henry Dale Fellowship jointly funded by the Wellcome Trust and the Royal Society (Grant Number 222546/Z/21/Z)’. RW is funded by the Darwin Trust. FB and AB are supported by ERC Consolidator Award 101002385 to AB.

## Conflict of interest

The authors declare that the research was conducted in the absence of any commercial or financial relationships that could be construed as a potential conflict of interest.

## Publisher’s note

All claims expressed in this article are solely those of the authors and do not necessarily represent those of their affiliated organizations, or those of the publisher, the editors and the reviewers. Any product that may be evaluated in this article, or claim that may be made by its manufacturer, is not guaranteed or endorsed by the publisher.
